# Product level dataset on embodied human appropriation of net primary production

**DOI:** 10.1016/j.dib.2023.109725

**Published:** 2023-10-29

**Authors:** Nicolas Roux, Lisa Kaufmann, Sarah Matej, Thomas Kastner, Alberte Bondeau, Helmut Haberl, Karlheinz Erb

**Affiliations:** a University of Natural Resources and Life Sciences, Vienna, Department of Economics and Social Sciences, Institute of Social Ecology, Schottenfeldgasse 29, 1070 Vienna, Austria; b Senckenberg Biodiversity and Climate Research Centre, Frankfurt am Main, Germany; c Aix Marseille Univ, Avignon Univ, CNRS, IRD, IMBE, Marseille, France

**Keywords:** Land use, Land-use intensity, Biomass, Trade, Agricultural products, Animal products, Food

## Abstract

This dataset includes data on the embodied human appropriation of net primary production (eHANPP) associated with products derived from agriculture and forestry. The human appropriation of net primary production (HANPP) is an indicator of changes in the yearly availability of biomass energy from photosynthesis that remains available in terrestrial ecosystems after harvest, under current land use, compared to the net primary production of the potential natural vegetation. HANPP is an indicator of land-use intensity that is relevant for biodiversity and biogeochemical cycles. The eHANPP indicator allocates HANPP to products and allows tracing trade flows from origin (the country where production takes place) to consumption (the country where products are consumed), thereby underpinning research into the telecouplings in global land use. The datasets described in this article trace eHANPP associated with the bilateral trade flows between 222 countries. It covers 161 primary crops, 13 primary animal products and 4 primary forestry products, as well as the end uses of these products for the years 1986 to 2013.

Specifications Table

**Table:** 

Subject	Environmental Science
Specific subject area	The production and consumption of agricultural and forestry commodities is putting pressure on ecosystems and biodiversity on land. These commodities are increasingly being traded internationally. The field of land use and international trade is quantifying the pressure on ecosystems and biodiversity embodied in the international trade and consumption of land-based commodities.
Data format	Processed data
Type of data	Table
Data collection	Production, trade and end uses of agricultural and forestry products were taken from FAOSTAT, combined with data on the potential state of ecosystems calculated using the LPJ_ml_ vegetation model, and factors from previous HANPP assessments to calculate the pressure on ecosystems embodied in global supply chains of biomass products.
Data source location	**Primary data sources (see section on Experimental design, materials and methods for more details):** FAOSTAT (downloaded on July 2021, see Table 1), Vegetation model (LPJmL), relying on CRU_TS4.03 historical climatology data (Harris et al. 2020) Land use maps [1] Cultivar maps [2], FAO Gridded Livestock of the world
Data accessibility	Repository name: Zenodo Data identification number: 10.5281/zenodo.8384359 Direct URL to data: https://doi.org/10.5281/zenodo.8384359 Instructions for accessing these data: .csv files can be opened with any data processing software. Version 1.0.0 of the data, available in the same link, contains the same data in .rds format, for lighter data handling, which can be opened in R, or in Python (with the “pyreadr” package).
Related research article	Roux, Nicolas, Lisa Kaufmann, Julia Le Noe, Sarah Matej, Perrine Laroche, Thomas Kastner, Alberte Bondeau, Helmut Haberl, and Karlheinz Erb. “Embodied HANPP of Feed and Animal Products: Tracing Pressure on Ecosystems along Trilateral Livestock Supply Chains 1986–2013.” Science of The Total Environment 851 (December 10, 2022): 158198. https://doi.org/10.1016/j.scitotenv.2022.158198.

## 1 Value of the Data

• The dataset on embodied Human Appropriation of Net Primary Production (eHANPP) reveals pressures on biodiversity and ecosystems, including their carbon balance, embodied in global supply chains of agricultural and forestry products.
• The data reveal trade flows between 222 countries, at the product level for 161 primary crops, 13 primary animal products and 4 primary forestry products, as well as the end uses of these products, for the years 1986 to 2013.
• The dataset is useful for consumption or flow-based accounts of the pressure on ecosystems, or to study effects of international trade on ecosystems and biodiversity, such as the displacement of ecological pressure to other countries, the global reallocation of agricultural production, etc.
• These data can be interesting for researchers, NGOs, consultancies or policy makers working on global supply chains and anthropogenic pressures on ecosystems.
• The data can be used to visualize the evolution of pressure on ecosystems linked to specific supply chains, to determine which commodities and trade flows are causing most pressure on ecosystems. It can also be used to compare the pressure on ecosystems linked to different end uses as animal products, vegetal human food, or materials and energy.
• Embodied HANPP Data are also of growing interest to quantify the overshoot of nations over planetary boundaries. See [3,4]

## 2 Data Description

For semantic explanations of the HANPP and eHANPP indicators, refer to [5].

File 1 [embodied_HANPP_all_uses_no_ap_trade_cl_gl_resid_by_feed_products.csv]

Contains HANPP embodied in the bilateral trade of primary crop products (excluding the residues used as feed), roughage and residues used as feed, by end uses (direct human food, animal feed, other uses), between 1986 and 2013. The HANPP embodied in bilateral trade flows of animal feed corresponds to the total trade flow from the country producing the feed to the country **producing** the animal product.

File 2 [embodied_HANPP_all_uses_incl_ap_all_cl_gl_resid_by_animal_products.csv]

Contains HANPP embodied in the bilateral trade of primary crop products, roughage and residues used as feed, combined to animal products, by end uses (direct human food, animal products, other uses), between 1986 and 2013. Unlike in File 1, the HANPP embodied in bilateral trade flows of animal products corresponds to the total trade flow from the feed producing country to the country finally **consuming** the animal product.

File 3 [trilateral_embodied_HANPP_cl_gl_resid_avg_2011_2013.csv]

Contains HANPP embodied in the trilateral trade (trade flows between countries producing feed, countries producing animal products, and countries consuming animal products) of feed crops, roughage and residues used as feed combined to animal products. Because of the size of the data, we only display the average data over the period 2011-2013 (for other years, please contact the authors). Each row represents the HANPP embodied in one combination of feed and animal product trade between a country producing feed, a country producing the animal product, and a country consuming the animal product (for example soybeans produced in Brazil, exported to Germany to produce pig meat, which itself is exported to and consumed in Austria).

FILE_NAME4 [embodied_HANPP_forest_IRW_and_WF.csv]

Contains HANPP embodied in the bilateral trade of 4 primary forestry products, between 1997 and 2013.

## 3 Experimental Design, Materials and Methods

FAOSTAT Data were accessed using the fenixservices API on July 2021 (code from https://github.com/martinbruckner/fabio_v1). Required FAOSTAT Data are described in Table 1. Note that the data structure and zip file names of FAOSTAT have been changed since we originally downloaded the data (e.g., production and commodity balances). The data downloadable through the FAOSTAT URLs in Table 1 do hence not exactly correspond to the original data used in this article.

**Table 1:** FAOSTAT Data required for the HANPP and eHANPP calculation.

Data	Bulk download Zip file	Filtered element or item
Crop production, crop harvested area	Production_Crops_E_All_Data_(Normalized).zip Current URL: https://fenixservices.fao.org/faostat/static/bulkdownloads/Production_Crops_Livestock_E_All_Data_(Normalized).zip	Production, Area harvested (Element)
Livestock production	Production_Livestock_E_All_Data_(Normalized).zip Current URL: https://fenixservices.fao.org/faostat/static/bulkdownloads/Production_Crops_Livestock_E_All_Data_(Normalized).zip	Production
Livestock animal numbers (Cattle and buffaloes)	Production_LivestockPrimary_E_All_Data_(Normalized).zip Current URL: https://fenixservices.fao.org/faostat/static/bulkdownloads/Production_Crops_Livestock_E_All_Data_(Normalized).zip	
Market feed from crops, end uses	CommodityBalances_Crops_E_All_Data_(Normalized).zip (Not fully reported anymore by the FAO)	
Market feed from livestock products	CommodityBalances_LivestockFish_E_All_Data_(Normalized).zip (Not fully reported anymore by the FAO)	
Forestry products production	Forestry_E_All_Data_(Normalized).zip URL: https://fenixservices.fao.org/faostat/static/bulkdownloads/Forestry_E_All_Data_(Normalized).zip	Production (Element)
Physically cropped area	Inputs_LandUse_E_All_Data_(Normalized).zip URL: https://fenixservices.fao.org/faostat/static/bulkdownloads/Inputs_LandUse_E_All_Data_(Normalized).zip	Cropland (Item)
Bilateral trade matrices agriculture	Trade_DetailedTradeMatrix_E_All_Data_(Normalized).zip URL: https://fenixservices.fao.org/faostat/static/bulkdownloads/Trade_DetailedTradeMatrix_E_All_Data_(Normalized).zip	
Bilateral trade matrices wood products	Forestry_Trade_Flows_E_All_Data_(Normalized).zip URL: https://fenixservices.fao.org/faostat/static/bulkdownloads/Forestry_Trade_Flows_E_All_Data_(Normalized).zip	

Countries that changed their border between 1986 and 2013 were the USSR, Belgium-Luxembourg, Yugoslavia, Serbia-Montenegro, Czechoslovakia, Ethiopia-Eritrea, Sudan. When factors were missing either for the subsequent countries, the factor of the former country was applied to the divided countries. When indices (degree of industrialization, biome, etc.) were missing for the former aggregated country, the index from the largest of the divided countries was taken. Spatially explicit data were shared according to country areas.

**Fig. 1:**
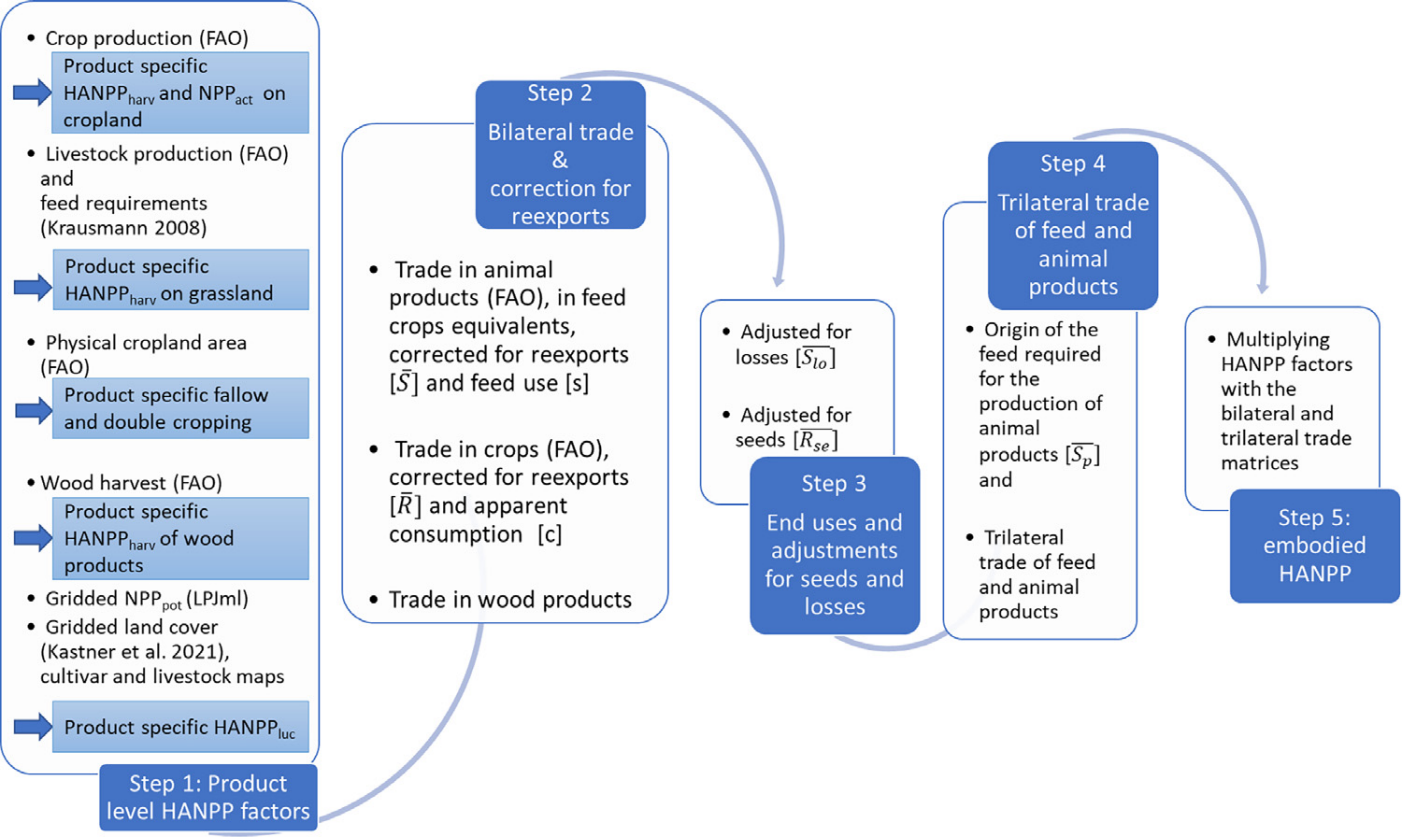
Calculation steps. Data sources are referred to in round brackets (…). Matrix or vector names are written in square brackets […]. NPP_pot_: potential Net Primary Production, NPP_act_: actual Net Primary Production, HANPP_harv_: HANPP induced by crop harvest or grazing, HANPP_luc_: HANPP induced by land use change.

Calculation steps are summarized in Fig. 1.

### 3.1 Step 1 Calculation of the HANPP factors per product

The product level HANPP calculation is similar in most steps to previous HANPP assessments [6,1,7], but keeping product disaggregation as much as possible.

#### 3.1.1 Step 1.1 HANPP_harv_ and NPP_act_ on Cropland

The calculations of HANPP_harv_ on cropland were similar to Kastner et al. [1] and Haberl et al. [6], except that crops were never aggregated.

$$\begin{array}{ccc} HANPP_{harv,\;crop} & = & \left(Crop\;production\;d.m.+used\;by\;products+unused\;by\;products\right)\\ & & \times shoot\;to\;total\;ratio \end{array}$$

We first converted crop production from fresh weight to dry matter using crop specific water content factors.

Cropproductiondrymatter=Cropproductionfreshweight×(1−watercontentfactor)

We calculated used by-products (out of which some are used as feed) and unused residues with specific multipliers (harvest indices) for each world region [8], differentiating annual and permanent crops.

$$\begin{array}{c} used\;by\;products=Crop\;production\;d.m.\;\times \;multiplier\;used\;by\;products\\ unused\;by\;products=Crop\;production\;d.m.\;\times \;multiplier\;unused\;by\;products \end{array}$$

Some annual crops, called $A_{0}$, had no harvest indices. These were attributed a multiplier of unused by-products defined as the average ratio of by-products of crops with harvest index ($A_{HI})$

$$\begin{array}{ccc} unused\;by\;products\;A_{0} & = & Crop\;production\;d.m.\;A_{0}\\ & & \times \frac{total\;used\;by\;products\;A_{HI}+total\;unused\;by\;products\;A_{HI}}{total\;crop\;production\;A_{HI}} \end{array}$$

Total above-ground $HANPP_{harv}$ was defined as:

$$Aboveground\;HANPP_{harv,\;cropland}=\;Crop\;production\;d.m.\;+\;used\;by\;products+unused\;by\;products\;$$

The total above and below-ground $HANPP_{harv}$ was calculated with a shoot to total ratio of ∼0.87 [9]. This yields the HANPP_harv_ on cropland at the product level.

$$HANPP_{harv,\;crop}\;=\;\frac{Aboveground\;HANPP_{harv,\;crop}}{\left(\frac{0.53}{0.53+0.08}\right)}$$

We calculated pre-harvest losses according to each country's degree of industrialization [10]. For permanent crops, the additional NPP remaining in ecosystems is estimated to 1.5 times the production [6]. The sum of the preharvest losses and the NPP remaining for permanent crops yields the NPPeco for each product.

$$NPP_{eco,\;crop}=pre\;harvest\;losses\times HANPP_{harv,\;crop}+1.5\;\times permanent\;crops$$

The sum of the HANPP_harv_ and the NPP_eco_ is the NPP_act_, at the product level.

$$NPP_{act,\;crop}=NPP_{eco,\;crop}+HANPP_{harv,\;crop}$$

#### 3.1.2 Step 1.2 HANPP_harv_ on Grassland and animal feed

The HANPP_harv_ on grassland was calculated using the grazing gap method, i.e. by calculating grazed biomass as the residual feed requirement after subtracting market feed and crop residues used as feed [11]. All equations are at the country and year level.

$$\begin{array}{ccc} HANPP_{harv,\;grassland} & = & total\;ruminant\;feed\;demand-1.5\;\times \;(Total\;market\;feed\\ & & -\;feed\;demand\;monogastrics-residues\;used\;as\;feed \end{array}$$

Daily feed (hay equivalent) demand for milk cows and beef cattle were taken from the linear functions between milk (beef) yield and feed intake, from Krausmann et al. [11].

$\text{Feed}\;\text{deman}d_{\text{beef}\;\text{and}\;\text{buffalo}\;\text{meat}\;}\left[\text{Mt}\;\text{dm}\;\text{hea}d^{-1}\;yr^{-1}\right]=\left(0.036361\;\times Carcass\;weight_{beef\;and\;buffalo\;meat}+1.702006\right)\times \;365$

$\text{Feed}\;\text{deman}d_{\text{milk}\;}\left[\text{Mt}\;\text{dm}\;\text{hea}d^{-1}\;yr^{-1}\right]=\left(0.00155\;\times Yield_{Milk}+4.8375\right)\times \;365$

Because the distinction between meat cattle and milk cattle is not straightforward, the feed demand from cows and cattle were set to the maximum between the two values (milk and meat).

$$\begin{array}{ccc} & & \text{Total}\;\text{feed}\;\text{deman}d_{\text{cattle}\;}\left[\text{Mt}\;\text{dm}\;yr^{-1}\right]=cattle\;heads\\ & & \;\;\;\times \max\left\{\text{Feed}\;\text{deman}d_{\text{beef}\;\text{and}\;\text{buffalo}\;\text{meat}\;};\;\text{Feed}\;\text{deman}d_{\text{milk}\;}\;\right\} \end{array}$$

Feed demand (hay equivalent) from other ruminants and grazing animals as horses (hereafter called ruminants or grazing animals) was calculated with a demand per head factor (Table 2). For sheep and goats, the factors are as well specific to the degree of industrialization.

$$Total\;feed\;demand_{other\;ruminants}\left[\text{Mt}\;\text{dm}\;yr^{-1}\right]\;=\;Feed\;demand_{other\;ruminants}\;*\;heads\;*\;365/10^{9}$$

**Table 2:** Factors for feed demand per head - other ruminants.

		Developing countries	Industrialized countries
Sheep	kg dm/head/day	1.0	1.5
Goats	kg dm/head/day	1.0	1.5
Horses	kg dm/head/day	10.0	
Asses	kg dm/head/day	6.0	
Mules	kg dm/head/day	6.0	
Camels	kg dm/head/day	10.0	

Feed demand for monogastrics was calculated via world region specific feed demand per unit product factors (Table 3).

$$\begin{array}{ccc} Total\;feed\;demand_{ruminants}\left[\text{Mt}\;\text{dm}\;yr^{-1}\right]\; & = & \;Total\;feed\;demand_{cattle}\\ & & +\;Total\;feed\;demand_{other\;ruminants} \end{array}$$

$$\begin{array}{ccc} & & Total\;feed\;demand_{monogastrics}\left[\text{Mt}\;\text{dm}\;yr^{-1}\right]\\ & & \;\;=\;\;Feed\;demand\;per\;product_{\;monogastrics}\;*\;production_{monogastric\;products} \end{array}$$

**Table 3:** Factors for feed demand per product: monogastrics.

Product	Unit	East Asia	East Europe	Latin America	North Africa and W. Asia	North America and Oc.	South and Central Asia	Subsaharan Africa	West Europe
Eggs Primary	kg feed/kg eggs	3	3	3	3	2.8	3.8	4	2.8
Meat, pig	kg feed DM/ kg meat FW	5	5	9	6	4	8	8.5	4
Meat, Poultry	kg feed/kg meat	4.3	4	3.6	4.4	3	5.1	5.5	3

We converted market feed from the commodity balances into dry matter, and allocated it in priority to monogastrics, as they cannot eat large quantities of roughage. Where market feed alone was not sufficient to cover the feed requirements of monogastrics, we assumed that the rest would have come from kitchen residues and other unformal sources, to which we do not allocate a HANPP rucksack.

$$\begin{array}{ccc} & & Market\;feed\;for\;monogastrics\;\left[\text{Mt}\;\text{dm}\;yr^{-1}\right]\\ & & \;\;=\;\text{min}\left\{Total\;feed\;demand_{monogastrics};\;Total\;market\;feed\;dm\right\} \end{array}$$

Market feed for ruminants was defined as the difference between total market feed (in dry matter) and the market feed allocated to monogastrics. For the grazing gap method, market feed for ruminants was multiplied by 1.5 to convert it into hay equivalents.

$$\begin{array}{ccc} & & Market\;feed\;for\;ruminants\;in\;hay\;equivalent\\ & & \;=\;\text{min}\{Total\;feed\;demand_{ruminants};\;(Total\;market\;feed\;dm\\ & & \;-\;\;Market\;feed\;for\;monogastrics)*1.5\} \end{array}$$

Used crop residues were taken from the cropland calculation, and multiplied by a country specific factor of residues used as feed for cereals and other crops. Residues used as feed were downscaled when these exceeded the remaining feed requirement of ruminants.

$$\begin{array}{ccc} Residues\;used\;as\;feed & = & \min\{cropland\;used\;by\;products\times share\;residues\;used\;as\;feed;\\ & & (Total\;feed\;demand_{ruminants}-Market\;feed\;for\;ruminants\\ & & in\;hay\;equivalent)\} \end{array}$$

The total feed from grazing, i.e. HANPP_harv_ on grassland, was calculated as the feed requirement of ruminants, neither covered by market feed nor by crop residues. Note that because the FAO does not report roughage from fodder crops (green maize etc.), the latter are embedded in the HANPP_harv_ on grassland.

$$\begin{array}{ccc} Total\;HANPP_{harv,\;grassland} & = & Total\;feed\;demand_{ruminants}-Market\;feed\;for\;ruminants\;in\;hay\;equivalent\\ & & -\;residues\;used\;as\;feed \end{array}$$

Previous calculation steps were similar to previous studies. For our purposes grazing has to be allocated to the various grazing animals. We hence calculated the shares of market feed, residues and roughage (grazing including roughage fodder crops) in the total feed demand of all ruminants.

$$Share_{feed\;type,\;ruminants}=\frac{feed\;type_{ruminants}}{Total\;feed\;demand_{ruminants}}$$

Where feedtype={Grazing;cropresidues;marketfeedinhayequivalent}

These shares are then multiplied to each ruminant's total feed demand. In other words, in each country, cattle, goats and sheep would be allocated the same share of market feed, crop residues and roughage.

$$feed\;type_{animal}=Share_{feed\;type,\;ruminants}\times Total\;feed\;demand_{animal}$$

Where animal={cattle;sheep;goats;horses;asses;mules;camels}

To retrieve the market feed in market feed equivalent, the market feed in hay equivalents must be divided by 1.5.

The shares of each market feed product within the total market feed were as well attributed to all animals. Therefore, within their respective market feed, pigs get the same share of soy cake or brans as do cattle.

$$Share_{feed\;product}=\frac{feed\;product}{Total\;market\;feed}$$

$$\begin{array}{ccc} Market\;feed_{feed\;product,animal\;or\;product} & = & Share_{feed\;product}\\ & & \times market\;feed\;in\;market\;feed\;equivalent_{animal\;or\;product} \end{array}$$

Where animalorproduct={cattle;sheep;goats;horses;asses;mules;camels;eggs;poultrymeat;pigmeat}

#### 3.1.3 Step 1.3 Fallow land and double cropping

We calculated the difference between the national cropland area under arable crops, and the total harvested area over all arable crops in each country.

$$\Delta area\;=\;Physical\;cropland\;area\;-\sum_{crops}area\;harvested$$

In countries and years where Δarea is negative, we consider it to be due to multiple cropping of arable crops (several harvesting periods on the same area).

$$multiple\;cropping\;factor=\;\frac{Total\;physical\;arable\;cropland\;area}{\sum_{arable\;crops}area\;harvested}$$

The multiple cropping factor is hence multiplied to the respective harvested area of each arable crop to obtain the physically cropped area.

$$Physical\;cropped\;area_{arable\;crop}=\;area\;harvested_{arable\;crop}\times multiple\;cropping\;factor$$

In countries and years where Δarea is positive, we set the difference to be fallow land.

Unlike previous HANPP assessments, we had to distribute this fallow to each arable crop, and opted for distributing it according to their respective physically cropped area.

$$fallow\;land_{arable\;crop}=total\;fallow\;land\times \frac{Physical\;cropped\;area_{arable\;crop}}{Total\;physical\;arable\;cropland\;area}$$

#### 3.1.4 Step 1.4 HANPP_harv_ of wood products

We first isolated the production of the four primary products, namely: industrial roundwood, coniferous; industrial roundwood, non-coniferous; Wood fuel, coniferous; Wood fuel, non-coniferous. Following previous HANPP assessments, wood volumes were converted in tonnes of dm biomass using the following wood density factors. We added the bark of the tree using bark factors, accounted for non-removed fellings with recovery rates, and for belowground NPP with a shoot over total ratio. For each of the primary products:

$$\text{HANP}P_{\text{harv},\;\text{wood}\;prod}=\frac{\text{wood}\;\text{market}\;\text{volume}\;\times \text{wood}\;\text{density}}{Bark\;factor\times recovery\;rate\times shoot\;over\;total}$$

#### 3.1.5 Step 1.5 NPP_pot_

Global gridded NPP_pot_ at half degree spatial resolution is provided yearly by the LPJmL dynamic global vegetation model (Schaphoff et al. [12]). The model is driven by the following input data: i) monthly temperature, precipitation, cloudiness, and rainy days from the CRU_TS4.03 historical climatology (Harris et al. [13]), covering the time period 1901 to 2018, ii) yearly atmospheric co2 concentration (Tans and Keeling, [14]), iii) soil texture (Nachtergaele et al., [15]).

The NPP_pot_ was attributed to countries and land cover types based on spatially explicit land cover data from Kastner et al., [1]. Country and land cover type specific NPP_pot_ data were then smoothed over the entire period, using a loess method, and set to 0 where the result was negative.

For the product level HANPP factors, NPP_pot_ has to be allocated to products and animals. NPP_pot_ on cropland was proportionally allocated to crop products based on their respective physically cropped area, including the fallow land. For permanent crops, the physical cropped area is the harvested area, and fallow land is null. The crop specific NPP_pot_ was adjusted based on the location of the cultivars, with factors from [16], which were calculated from Monfreda et al. cultivar maps for the year 2000 [2]. The total was scaled to the new total NPP_pot_ values.

$$NPP_{pot,crop}=\left(Physical\;cropped\;area_{crop}+fallow\;land_{crop}\right)\times NPP_{pot,crop}\;per\;ha_{Kastner\;et\;al.}\times \frac{NPP_{pot,cropland}}{\sum_{crops}\left(\left(Physical\;cropped\;area_{crop}+fallow\;land_{crop}\right)\times NPP_{pot,crop}\;per\;ha_{Kastner\;et\;al.}\right)}$$

In countries where factors from Kastner et al. [16] were missing, we allocated the NPP_pot_ proportionally to the physically cropped area.

$$NPP_{pot,crop}=\frac{Physical\;cropped\;area_{crop}+fallow\;land_{crop}}{total\;physical\;cropped\;area}\times Total\;NPP_{pot,cropland}$$

NPP_pot_ on fallow was isolated by multiplying crop and country specific NPP_pot_ per hectare values, to the corresponding fallow area.

$$NPP_{pot,crop,fallow}=\frac{NPP_{pot,crop}}{Physical\;cropped\;area_{crop}+fallow\;land_{crop}}\times fallow\;land_{crop}$$

NPP_pot_ on grassland was first distributed to minor grazing animals (asses, mules, camels) proportionally to their respective share in the total HANPP_harv_ on grassland in each country.

$$NPP_{pot,grass,minor\;grazing\;animals}=\frac{HANPP_{harv,grass,animal}}{Total\;HANPP_{harv,\;grassland}}\times Total\;NPP_{pot,grassland}$$

For cattle and buffaloes, goats, horses, and sheep (hereafter called major grazing animals), we proceeded to a further adjustment of the NPP_pot_, to consider that certain animals (often cattle) graze on more productive land than others (often sheep and goats). We calculated HANPP_harv_ per head, and combined it to the dasymetric (DA) Gridded Livestock of the World (GLW) spatially explicit dataset from the FAO (https://www.fao.org/livestock-systems/global-distributions/en/), for the year 2010, to globally map the HANPP_harv_ on grassland for each animal for all years.

Within each pixel:

$$HANPP_{harv,grass,animal}\;in\;pixel=\;\#animals\;in\;pixel\times \frac{HANPP_{harv,grass,animal}}{Total\;\#animals_{Gridded\;livestock}}$$

Where animal={cattleandbuffaloes;goats;horses;sheep}

Summing the HANPP_harv_ maps of all animals, we gridded the share of each animal's HANPP_harv_ in total HANPP_harv_ on grassland. We combined the gridded shares of HANPP_harv_ to the gridded NPP_pot_ from grassland of major grazing animals, and multiplied the NPP_pot_ of grassland by each animal's share of HANPP_harv_ from grazing within each pixel, to obtain the animal specific NPP_pot_.

$$NPP_{pot,animal}=\left(Total\;NPP_{pot,grass}-Total\;NPP_{pot,grass,minor\;grazing\;animals}\right)\times share\;NPP_{pot,animal}$$

$$NPP_{pot,animal}=\left(Total\;NPP_{pot,grass}-Total\;NPP_{pot,grass,minor\;grazing\;animals}\right)\times share\;NPP_{pot,animal}$$

For countries small countries that were not in the raster, we kept the proportionally allocated values (as for minor grazing animals).

In some countries, the HANPP_harv_ of wood products exceeded the NPP_pot_ in forests. We assumed this is because wood products, especially wood fuels, may be harvested on other land cover types than forests, for example bush lands and savannahs. We hence adjusted the HANPP_harv_ of wood products, to isolate the HANPP_harv_ in forests only. We first removed the excessive HANPP_harv_ from the HANPP_harv_ of wood fuel (wf). In countries where this was still not sufficient, i.e. where the HANPP_harv_ of industrial roundwood (ir) alone was above the NPP_pot_ of forests, we removed the remaining excess from the HANPP_harv_ of industrial roundwood.

$$If\;Total\;HANPP_{harv,wood}>NPP_{pot,forest}:$$

$$\begin{array}{ccc} HANPP_{harv,\;wf,\;\left\{coniferous;\;non\;coniferous\right\},forest} & = & HANPP_{harv,\;wf,\left\{coniferous;\;non\;coniferous\right\}}\\ & & -\;share_{wf,\left\{coniferous;\;non\;coniferous\right\}}\\ & & \times \left(Total\;HANPP_{harv,wood}-NPP_{pot,forest}\right) \end{array}$$

Where

$$share_{wf,\left\{coniferous;\;non\;coniferous\right\}}=\frac{HANPP_{harv,wf,\left\{coniferous;\;non\;coniferous\right\}}}{HANPP_{harv,\;wf,coniferous}+HANPP_{harv,wf,non\;coniferous}}$$

Is the share of coniferous and non-coniferous wood in the total HANPP_harv_ of wood fuel.

$$If\;Total\;HANPP_{harv,ir}>NPP_{pot,forest}:$$

$$HANPP_{harv,\;wf,forest}=0$$

$$\begin{array}{ccc} HANPP_{harv,\;ir,\;\left\{coniferous;\;non\;coniferous\right\},forest} & = & HANPP_{harv,\;ir,\left\{coniferous;\;non\;coniferous\right\}}\\ & & -\;share_{ir,\left\{coniferous;\;non\;coniferous\right\}}\\ & & \times \left(Total\;HANPP_{harv,wood}-NPP_{pot,forest}-HANPP_{harv,wf}\right) \end{array}$$

Where $share_{ir,\{coniferous;\;non\;coniferous\}}$ is the share of coniferous and non-coniferous wood in the total HANPP_harv_ of industrial roundwood.

We can now allocate the NPP_pot_ in forests proportionally to the HANPP_harv_ in forest of the four primary wood products.

$$NPP_{pot,\;forest,prod}=NPP_{pot,forest}\times \frac{HANPP_{harv,\;prod,forest}}{Total\;HANPP_{harv,forest}}$$

#### 3.1.6 Step 1.6 HANPP_luc_ and HANPP

HANPP_luc_ on fallow was calculated by multiplying the NPP_pot_ on fallow with factors from [7]. NPP_act_ on fallow is hence the difference between the NPP_pot_ on fallow and the HANPP_luc_ on fallow. The total NPP_act_ on cropland is the sum of the NPP_act_ cropland and the NPP_act_ on fallow. HANPP_luc_ on cropland is the difference between NPP_pot_ on cropland and total NPP_act_ on cropland. The crop specific HANPP on cropland is the sum of the crop specific HANPP_harv_ and HANPP_luc_ on cropland.

$$\begin{array}{c} Total\;NPP_{act,crop}=NPP_{act,crop}+NPP_{pot,crop,fallow}\times \left(1-HANPP_{luc,fallow}\;per\;NPP_{pot}\right)\\ HANPP_{luc,crop}=NPP_{pot,crop}-NPP_{act,crop}\\ HANPP_{crop}=HANPP_{harv,crop}+HANPP_{luc,crop} \end{array}$$

Country specific average HANPP_luc_ per NPP_pot_ on grassland were taken from [7], and multiplied to the animal and country specific NPP_pot_ values. The animal specific HANPP on grassland is the sum of the animal specific HANPP_harv_ and HANPP_luc_ on grassland. NPP_act_ on grassland is the NPP_pot_ minus HANPP_luc_ on grassland. NPP_eco_ on grassland is the NPP_act_ minus HANPP_harv_ on grassland. Where the NPP_eco_ on grassland was below 5% of the NPP_act_, we adjusted the NPP_act_ upwards (and therefore the HANPP_luc_ downwards) as to have a NPP_eco_ of minimum 5% of NPP_act_, while keeping the HANPP_harv_ constant. This can be thought of as increasing grassland productivity through fertilization, as it is done in several intensive grazing systems (Netherlands, etc.).

For all animals, countries and years where $NPP_{eco}<0.05\;\times NPP_{act}$:

$$\begin{array}{c} NPP_{eco,grass,adjusted}=\frac{0.05}{0.95}\times HANPP_{harv,grass}\\ NPP_{act,grass,adjusted}=NPP_{eco,grass,adjusted}+HANPP_{harv,grass}\\ HANPP_{luc,grass,adjusted}=NPP_{pot}-NPP_{act,grass,adjusted}\\ HANPP_{grass,adjusted}=HANPP_{harv,grass}+HANPP_{luc,grass,adjusted} \end{array}$$

We did not allocate any HANPP_luc_ to crop residues used as feed. Therefore, the only HANPP allocated to crop residues used as feed was the mass of these residues (HANPP_harv_). This comes from the assumption that the land use change occurred primarily for the main product of the corresponding crop (for example wheat), and not for its by-product (straw).

For forestry products, the HANPP_luc_ is set to 0. NPP_act_ is hence equal to NPP_pot_ and NPP_eco_ equals NPP_pot_ minus HANPP_harv,forest_ (i.e. excluding the HANPP_harv_ exceeding the NPP_pot_ in forest), all at the primary product level.

#### 3.1.7 Step 1.7 factors used to calculate HANPP / product ratios

For cropland, we calculated the factors per tonne of crop by first removing the residues used as feed from the HANPP_harv_ and the HANPP on cropland, and dividing the respective value by the crop production quantity (in dry matter).

For grassland and crop residues used as feed, the allocation to animal products requires one further step, for grazing animals, namely the attribution to milk or meat (we did not consider other products as leather and materials, hence overestimating the rucksack of meat and milk). For grazing animals, we allocate HANPP values according to the number of heads reported for the production of milk or meat respectively.

$$share\;prod\;=\frac{\#animals_{prod}}{\#animals}$$

Where prod={milk;meat} and animals={cattle;sheep;goat}

For horses, asses, mules, and camels, we did not allocate the HANPP to the reported milk and meat, as we assumed that these animals are mainly kept for services and leisure, and we do not know how many animals are grown exclusively for products.

We finally calculate NPP and HANPP values per tonne of product, by dividing by the production quantities of the respective animal products.

For wood products, factors per tonne of product were calculated by dividing NPP and HANPP values by the production of wood in dry matter. Note that these values do not include the HANPP exceed the NPP_pot_ in forest, which are saved separately.

This finishes the HANPP calculation at the product level. Product level HANPP data can be visualized interactively and downloaded under https://ijsadihsadoaisjd.shinyapps.io/shiny_app_hanpp_all_lu_types/

### 3.2 Step 2: Bilateral trade and correction for reexports

#### 3.2.1 Step 2.1 Crops, animal products, and wood products

The calculation of the trilateral trade data relies on the same procedure as the one described in [[17], [18], [19]]. In this method, bilateral trade matrices are built, based on the reports from the importing countries. When the data from the importing country were missing, they were filled with data reported by exporting countries, yielding bilateral trade matrices between 222 countries.

283 traded vegetal products were converted to 159 primary crops, based on their dry matter content. Similarly, bilateral trade matrices were built for 66 traded animal products, converted to 13 primary animal products.

$$bilateral\;trade_{primary,dm}=\sum_{prod}bilateral\;trade_{prod,\;primary,fw}\times \left(1-water\;content\;factor\right)$$

Where prod,primary=allsecondaryproductsassociatedtoagivenprimaryproduct

Special treatment was given to sugar crops, often reported as “sugar refined”, which have to be split into sugar beet and sugar cane. We hence calculated the available supply of sugar beet and cane in each country, by correcting a first time for reexports, as described in [19], solely for sugar beet and cane. We then split the trade flows of refined sugar proportionally to the apparent consumption of sugar beet and cane in each country exporting refined sugar.

$$sugar\;refined_{\left\{beat;\;cane\right\}}=\frac{apparent\;consumption_{\left\{beat;cane\right\}}}{apparent\;consumption_{beat}+apparent\;consumption_{cane}}\times sugar\;refined$$

We could then correct for reexports for all primary crops as described on [19]. The difference of 0.3% between total production and the results of the corrected trade matrices was allocated to domestic consumption. Following the notation of [19], we hereafter call $\bar{R}$ the (n×n) sized bilateral trade matrix for a given crop, corrected for reexports, where ${\bar{R}}_{ij}$ is the the apparent consumption of country i originating from country j.

Despite the reported milk and meat products for Asses, Camels, Horses, Mules, we decided to neglect the production and international trade of these products, given that the large share of these animals is not used for their products. For examples, horses are likely rather used for horse riding than for horse meat. We hence considered that all the footprint of these animals was domestic.

For wood products, we followed the methodology of [20]. This method is similar to Kastner et al. [19], but corrects trade flows for the recovered paper and pulp, such that the trade flows reflect only exports of virgin (non-recovered) paper. As bilateral trade of wood fuel is small and not reported, we allocated all wood fuel to domestic consumption.

#### 3.2.2 Step 2.2 Animal products trade in primary crops equivalents

In order to calculate the matrix of livestock product trade in feed crop equivalents $\bar{S}$, we need to multiply the bilateral trade matrices of animal products by factors of feed crops per tonne of each animal product. Feed products per tonne of animal product were calculated previously for the grazing gap method. However, feed products have to be converted into primary crops. We hence built equivalence tables between the feed products reported in the FAO commodity balances and the primary crops. For example, some categories as brans can correspond to various crops (wheat, rice, etc.). Commodity balances products were hence distributed to the primary crops found in the description column of the FAOSTAT definitions, based on the respective apparent consumption of each crop calculated at the previous step through the bilateral trade matrices of crop products. Therefore, if corrected for imports and exports, out of all the crop products reported in the definition of brans, a country had an apparent consumption of 70 tonnes of wheat and 30 tonnes of rice, we allocated 70% of the brans to wheat and 30% to rice.

$$\begin{array}{ccc} feed\;crop\;embodied\;in\;animal\;product\;trade & = & AP\;trade\times CBS\;feed\;product\;per\;tonne\;of\;animal\\ & & product\times share\;of\;primary\;crops\;in\;feed\;products \end{array}$$

where

$$share\;of\;primary\;crops\;in\;feed\;products=\frac{apparent\;consumption_{crop}}{\sum_{crops\;in\;feed\;prod}apparent\;consumption_{crop}}$$

Animal products used as feed (e.g. cow milk or fish meal fed to other animals) were neglected from this step. Consequently, any animal product used as feed was dealt with as if it would have been finally consumed in the country where it was used to feed other animals, hence underestimating the international footprint of animal products. Hereafter, we call $\bar{S}$ the matrix of a given animal product, in feed crops equivalent.

### 3.3 Step 3: Calculating the final uses, adjusting for seeds and losses

We linked the trade data of crops to their final uses (feed, direct human food and other uses), adjusting for seed use and losses, based on the commodity balances (CBS) from the FAO. Again, the first step was to allocate all products from the commodity balances to primary crops according to apparent consumption, using the method described earlier (for the feed products). Sugar refined was again replaced by sugar cane and sugar beet.

$$\begin{array}{ccc} final\;use_{primary\;crop} & = & \sum_{cbs\;products}final\;use\;in\;dry\;matter_{cbs\;product}\\ & & \times \frac{apparent\;consumption_{primary\;crop}}{\sum_{primary\;crops\;in\;cbs\;product}apparent\;consumption_{primary\;crop}} \end{array}$$

For example, if 10 tonnes of brans are used as feed in a country, and that country apparently consumes 30 tonnes of wheat and 70 tonnes of maize, and that apart from brans 60 tonnes of maize are used for animal feed, then $10\;\times \frac{70}{70+30}+60=67$ tonnes of maize are used as feed in that country. The final use “processing” was removed to avoid double counting.

Trade matrices are to be adjusted to seed use, as the consumption of a crop in a given country should include the quantity of seeds globally used to produce that crop, but exclude the amount of that crop which this country is using for its own seeds. We adjusted trade matrices for seed use, by adding seeds to the production (and exports), and eventually removing them from the consumption (and imports). Ideally, one should add seeds from the previous year to the production, and remove seeds from that year from the consumption (as these are to be used the following year). However, for calculation ease (especially in the case of countries whose borders changed) we decided to assume that all seeds were used the same year. The adjustment for seeds does hence not affect the global quantity of crops, but solely the trade patterns.

Call $v=1+\frac{seeds\;in\;producing\;country}{production\;in\;producing\;country}\;$the vector adjusting production and exports for seed use, and $\hat{v}$ the corresponding diagonal matrix.

Call $w=1-\frac{seeds\;in\;consuming\;country}{consumption\;in\;consuming\;country\;(including\;seeds)}$ the vector adjusting consumption and imports for seed use, and $\hat{w}$ the corresponding diagonal matrix.

Then we calculated the trade matrix adjusted for seeds:

$$\overset{︸}{R_{se}}=w\times \bar{R}\times v$$

We allocated losses to feed, food and others (for simplicity, we assumed no losses in seeds). We calculated a loss adjustment factor in each country for each crop, and applied this factor to the quantity of feed, food and other uses.

$$\begin{array}{ccc} final\;use\;adjusted\;for\;losses_{primary\;crop} & = & \left(1+\;\frac{\text{losse}s_{primary\;crop}}{\sum_{\left\{\text{feed};\;\text{food};\;\text{other}\;\text{uses}\right\}}final\;uses_{primary\;crop}}\right)\\ & & \times final\;use_{primary\;crop} \end{array}$$

We eventually calculated the share (including losses) of the final end uses feed, food and other uses.

$$\begin{array}{ccc} & & share\;final\;use\;adjusted\;for\;losses_{primary\;crop}\\ & & \;=\frac{final\;use\;adjusted\;for\;losses_{primary\;crop}}{\sum_{\left\{\text{feed};\;\text{food};\;\text{other}\;\text{uses}\right\}}final\;uses\;adjusted\;for\;losses_{primary\;crop}} \end{array}$$

By multiplying the values of  $\overset{︸}{R_{se}}$ by the shares of the final uses adjusted for losses, we obtained the trade data of crops by end uses. Where data for final uses were not available, we attributed the data to the category “unknown final use”.

We calculated $\overset{︸}{S_{lo}}$ the matrix of a given animal product, in feed crops equivalent, adjusted for the losses in feed crops.

$$\begin{array}{ccc} & & feed\;crop\;embodied\;in\;animal\;product\;trade\;adjusted\;for\;losses\\ & & \;=feed\;crop\;embodied\;in\;animal\;product\;trade\\ & & \;\times {\left(1+\frac{\text{losses}}{\sum_{\left\{\text{feed};\;\text{food};\;\text{other}\;\text{uses}\right\}}final\;uses}\right)}_{producing\;country} \end{array}$$

We omitted the differences in final uses of animal products (e.g. leather).

### 3.4 Step 4: Origin of the feed for the production and consumption of livestock products

$$c=\;\overset{︸}{R_{se}}\times i$$

is the row sum of$\;\overset{︸}{R_{se}}$, and hence the vector of apparent consumption adjusted for seed use, with i a summation vector of 1. $\hat{c}$ is the diagonal matrix with the entries of c.

$${\hat{c}}^{-1}\times \overset{︸}{R_{se}}$$

is the mix of origin for that crop adjusted for seed use.

$$s=i^{\prime }\;\times \overset{︸}{S_{lo}}$$

is the feed embodied in the production of that animal product, adjusted for losses. $\hat{s}$ is the diagonal matrix with the entries of s.

Then $\overset{︸}{S_{p}}=\hat{s}\times \;{\hat{c}}^{-1}\times \overset{︸}{R_{se}}$ is the trade matrix reflecting the origin of the feed required for the production of that animal product. ${\overset{︸}{S_{p}}}_{ij}$ is the feed originating from country j used to produce an animal product in country i.

$$\overset{︸}{S\_c}=\overset{︸}{S_{lo}}\times \;{\hat{c}}^{-1}\times \overset{︸}{R_{se}}$$

is the trade matrix reflecting the origin of the feed embodied in the final consumption of that animal product. ${\overset{︸}{S_{c}}}_{ij}$ is the feed originating from country j embodied in the final consumption of an animal product in country i.

We calculated $\overset{︸}{S_{p}}$ and$\;\overset{︸}{S_{c}}$ for all crops and animal products. The level of disaggregation would show the quantity of a given crop originating from country A embodied in the production or in the final consumption of an animal product in country B (for example the quantity of soybeans originating from Brazil embodied in the pig meat production or final consumption in Germany).

$$\overset{︸}{S_{lo}}\times {\hat{s}}^{-1}$$

is the mix of final destination of an animal product in each country's production of that animal product. By multiplying the entries of $\overset{︸}{S_{p}}$ by the shares of each final destination of the animal product, we calculated the amount of each crop originating in country A, used to produce animal products in country B, which are eventually consumed in country C (for example, the quantity of Brazilian soybeans embodied in pig meat produced in Germany and exported to China).

### 3.5 Step 5: Calculate the HANPP embodied in trade

For eHANPP from cropland and forestry products, we calculated the HANPP (and other HANPP components, as HANPP_harv_, HANPP_luc_ or physically cropped area) embodied in the final use of crops for food and other uses, as well as for animal products production and final consumption, by multiplying the HANPP factors calculated in step 1.7, to the corresponding trade matrices.

For eHANPP from grassland and crop residues used as feed, we applied the HANPP factors directly to the trade matrices of animal products. For simplicity, we omitted international trade in crop residues (straws) and roughage (grass and fodder crops). The HANPP embodied in the production of animal products was hence set to the HANPP calculated in step 1. As explained earlier, we as well set the HANPP embodied in the “final consumption” of asses, camels, horses and mules equal to their HANPP in that given country, reflecting the idea that most of the feed and grazing of these animals is not meant to produce meat and milk, but rather to provide services (transport, work or leisure) enjoyed domestically.

Data handling were performed using the R software. The calculation explained above, combining raw data and factors from various sources, makes this dataset unique. All code can be found attached to this article. The final eHANPP data files are publicly available under https://doi.org/10.5281/zenodo.8384359

## 4 Limitations

See section 4.3. “Limitations” in the related research article [21] https://doi.org/10.1016/j.scitotenv.2022.158198.

## Ethics Statement

the authors have read and follow the ethical requirements for publication in Data in Brief and confirming that the current work does not involve human subjects, animal experiments, or any data collected from social media platforms.

## CRediT authorship contribution statement

**Nicolas Roux:** Conceptualization, Methodology, Writing – original draft. **Lisa Kaufmann:** Conceptualization, Methodology, Writing – review & editing, Supervision. **Sarah Matej:** Conceptualization, Writing – review & editing. **Thomas Kastner:** Methodology, Writing – review & editing. **Alberte Bondeau:** Formal analysis, Writing – review & editing. **Helmut Haberl:** Conceptualization, Writing – review & editing, Supervision, Project administration. **Karlheinz Erb:** Conceptualization, Writing – review & editing, Supervision.

## Data Availability

Product level dataset on embodied Human Appropriation of Net Primary Production (Original data) (Zenodo)

## Appendix Supplementary materials

Image, application 1
